# Emotion Recognition Using a Reduced Set of EEG Channels Based on Holographic Feature Maps

**DOI:** 10.3390/s22093248

**Published:** 2022-04-23

**Authors:** Ante Topic, Mladen Russo, Maja Stella, Matko Saric

**Affiliations:** Faculty of Electrical Engineering, Mechanical Engineering and Naval Architecture, University of Split, 21000 Split, Croatia; mrusso@fesb.hr (M.R.); mstella@fesb.hr (M.S.); msaric@fesb.hr (M.S.)

**Keywords:** electroencephalogram, Brain-Computer Interface, ReliefF, Neighborhood Component Analysis, deep learning, computer-generated holography, gender specific emotion recognition, valence-arousal-dominance model

## Abstract

An important function of the construction of the Brain-Computer Interface (BCI) device is the development of a model that is able to recognize emotions from electroencephalogram (EEG) signals. Research in this area is very challenging because the EEG signal is non-stationary, non-linear, and contains a lot of noise due to artifacts caused by muscle activity and poor electrode contact. EEG signals are recorded with non-invasive wearable devices using a large number of electrodes, which increase the dimensionality and, thereby, also the computational complexity of EEG data. It also reduces the level of comfort of the subjects. This paper implements our holographic features, investigates electrode selection, and uses the most relevant channels to maximize model accuracy. The ReliefF and Neighborhood Component Analysis (NCA) methods were used to select the optimal electrodes. Verification was performed on four publicly available datasets. Our holographic feature maps were constructed using computer-generated holography (CGH) based on the values of signal characteristics displayed in space. The resulting 2D maps are the input to the Convolutional Neural Network (CNN), which serves as a feature extraction method. This methodology uses a reduced set of electrodes, which are different between men and women, and obtains state-of-the-art results in a three-dimensional emotional space. The experimental results show that the channel selection methods improve emotion recognition rates significantly with an accuracy of 90.76% for valence, 92.92% for arousal, and 92.97% for dominance.

## 1. Introduction

Given the importance of emotions in everyday human life, emotion recognition is a key function in the construction of Human-Computer Interaction (HCI) devices. Emotion is a physiological state that reflects human feelings, thoughts, and behavior and plays an extremely important role in the way humans create perceptions and make rational decisions. Recognition of emotions can be achieved by analyzing non-physiological signals such as speech [1], facial expression [2], and body posture [3]. However, these signals are often difficult to recognize. The application of wearable sensors may overcome this problem. Physiological signals that can be collected using these wearable sensors are photoplethysmogram, electrocardiogram (ECG), electrodermal activity, electromyogram, and electroencephalogram (EEG). Data collected via wearable sensors are used as inputs for analyzing emotions. These data are often considered to be more reliable because they are based on unconscious body changes, which are controlled by the sympathetic nervous system, which makes them difficult to manipulate.

One of the key steps toward emotional intelligence is the recognition of emotions from brain signals because the EEG signal can directly detect brain dynamics responding to different emotional states. The development of reliable and accurate Brain-Computer Interface systems has been a topic of numerous pieces of research in the last few decades [4,5]. BCI systems are based on EEG signals that represent the voltage difference between the active and reference electrodes over time. The recorded brain signals have various disadvantages caused by artifacts due to muscular activity and poor electrode contact. The EEG signal is non-stationary, non-linear in nature, and contains a lot of noise, which is one of the main challenges for researchers in this field. How to effectively process EEG signals, single out critical features, and build a model for extracting and classifying emotions is another major challenge.

The frontal, parietal, temporal, and occipital are the four lobes of the cerebral cortex. EEG devices can be categorized according to the number of electrodes into a low-resolution group (1–32), a medium-resolution group (33–128), and a high-resolution group (>128) [6]. An international 10–20 system [7] of placing electrodes on the scalp is commonly used in which the locations of each electrode are known by its relative location. By placing EEG electrodes on the most optimal regions of the brain from the perspective of recognizing emotions, the number of electrodes can be reduced and the level, of comfort in people wearing a sensor can be increased. It is precisely this problem that researchers have been focusing on recently to enable the use of the EEG headset in real-world practice usage.

From the emotion recognition point of view, the optimal channels for EEG-based emotion recognition are not fully determined. Therefore, the motivation for this work is to give another view of the aforementioned challenges that researchers face today. In particular, we focused on exploring the most relevant channels that consequently reduce computational complexity and mitigate noise in the process of recognizing human emotional states. Additional research was undertaken between men and women using previously selected channels. For this purpose, we created R-HOLO-FM and N-HOLO-FM holographic feature maps created from the various characteristics of the EEG signals utilizing ReliefF and NCA, respectively. Deep learning and machine learning techniques were used to extract features and classify them into a three-dimensional space of valence, arousal, and dominance. We conducted our experiments on four publicly available emotion datasets: DEAP, DREAMER, AMIGOS, and SEED. By using the proposed approach, we have achieved results that outperform most studies that have used comparable methods. This paper investigates the use of ReliefF and NCA to select the most optimal channels in the process of recognizing emotions from the EEG signals and provides insight into the classification of emotions in 3D emotional space from the EEG signals depending on gender.

The rest of this paper is structured as follows. Section 2 describes the related work. Section 3 gives a description of the used datasets, the 3D emotional space, and the signal characteristics. The procedures for creating the holographic feature maps with a reduced set of EEG channels and model construction are described in Section 4. Furthermore, the selection of the most optimal channels by gender, as well as by individual dataset, is described in the same chapter. The results are discussed in Section 5, and the conclusion is presented in Section 6.

## 2. Related Work

Today’s EEG sensors use wearable devices to collect raw data and wireless data transmission, even for web-based applications [8]. Although signals from all EEG channels are traditionally used for analysis, electrode set reduction is commonly investigated mainly for the use of EEG headsets in daily life. The five main categories of channel selection algorithms for EEG signal processing are filtering, wrapping, embedded, hybrid, and human-based techniques [9]. In terms of emotion classification, channel selection methods can be categorized as filtering and wrapper techniques. Numerous papers show that it is possible to reduce the number of electrodes without a drastic drop in performance. For example, the authors in [10] propose a method based on synchronization likelihood (SL) and anatomical knowledge for the automatic selection of optimal electrodes. They reduced the number of electrodes from 64 to five with a slight loss in the classification accuracy rate. This was also confirmed earlier by the authors of [11], who showed that the frontal pairs of channels give a better result than the channels located in the other head area by obtaining a classification accuracy of 85.41%. Based on the above, the authors in [12] selected 12 channels located near the frontal lobe.

In general, emotions can be classified into discrete values [13] or in dimensional emotional space, thus distinguishing between the two-dimensional Valence-Arousal [14] and the three-dimensional Valence-Arousal-Dominance space [15] we used in this research. An example of a study focused on optimizing EEG channels and classifying emotions into nine discrete values: happy, pleasant, relaxed, calm, excited, neutral, distressed, miserable, and depressed, was performed by [16] on the DEAP dataset. Using the zero-time windowing method, the authors extracted the current spectral information of each of the four bands: alpha, beta, delta, and gamma, for the adaptive number of channels per participant (from two to ten) and achieved an accuracy of 89.33%. Some of the reported studies used only a few channels (<5), defining them from previous studies that experimentally showed which brain lobes are active during emotion recognition. A similar study was performed in [17], where the data from 16 participants was recorded and classified into six basic emotions using Fp1, Fp2, F3, and F4 positions for the EEG acquisition. The HAF-HOC (Hybrid Adaptive Filtering–Higher Order Crossings). The methodology applied provides classification rates of up to 85.17%. In terms of channel selection, the study [18] obtained a pool of eight electrodes: AF3, AF4, F3, F4, F7, F8, T7, and T8 and achieved an average accuracy of 87.50% by classifying emotions in four emotional states: amused, disgusted, sad, and neutral.

Zhang et al. [19] investigated a channel selection scheme based on ReliefF in EEG-based emotion recognition and showed that the channels involved in the classification task could be dramatically reduced with an acceptable loss of accuracy. They systematically investigated different strategies to select the best channels, classifying four emotional states (joy, fear, sadness, and relaxation). For instance, they explored Subject-Dependent and Subject-Independent Channel Selection by applying the mean-ReliefF-channel-selection (MRCS) method with an SVM classifier. Nineteen channels based on the DEAP dataset achieved the best classification accuracy of 59.13%. Another use of the ReliefF algorithm based on the DEAP dataset for channel selection was presented in [20]. They used the Relief-FGSBS (Floating Generalized Sequential Backward Selection) EEG channel selection method and experimented on a self-collected data set and on the publicly available DEAP dataset. The results of the research showed that the most optimal EEG channels are mainly located in the frontal lobe and the posterior occipital region. The SVM used to classify emotions had 10-channel EEG signals and achieved an average classification accuracy of 91.31%.

Channels can be selected either manually based on previous research or by applying methods to optimize the selection of the most relevant channels, which are usually the channels with the highest weighting factors. Li et al. [21] concluded that the classification accuracy of emotional states increases with the number of channels. They investigated the EEG classification with 10, 14, 18, and 32 channels of the DEAP dataset, where the channels were selected based on the experience of other authors. Entropy and energy were calculated as characteristics on four frequency bands decomposed using discrete wavelet transformation. The classification was performed into valence and arousal dimensions using k-Nearest Neighbor (kNN). A similar technique for decomposing EEG signals into gamma, beta, alpha, and theta subbands, and the application of various classifiers, including kNN, was used in [22]. Spectral features were extracted from ten channels from the frontal cortex and obtained state-of-the-art results on the beta subband in the 2D emotional model. The research undertaken in [23] applied the same pool of electrodes as in [22] but obtained slightly worse results in both valence and arousal dimensions. Another approach in which channels are selected manually is [24], in which the five EEG channels having the highest performance in emotion recognition: P3, FC2, AF3, O1, and Fp1, were used to investigate valence, i.e., positive and negative emotions. The authors demonstrated the superiority of the MultiLayer Perceptron Neural Network (MLPNN) method over the SVM.

Article [25] proposes to use information from the time-frequency domain using normalized mutual information (NMI) as a channel selection method to select an optimal subset of EEG channels. The Short-time Fourier transform (STFT) is adopted to capture the EEG spectrogram, which is actually a matrix that reflects the energy distribution of the signal at different frequencies. The channels were selected by connection matrix analysis using an inter-channel connection matrix with NMI. The challenge with the proposed methods is that most of the tests were based on individual data sets, which makes them difficult to compare. The papers [26,27,28] verified their proposed methods on two or three datasets, while this paper conducts research on four publicly available datasets. The study performed in [26] examined which brain areas are active during emotional processing and concluded that out of a total of eight channels used, they are the frontal (F8), parietal (P7), and temporal (T8 and T7). Furthermore, under emotional stimulation, the areas around the AF4, F8, O1, P7, T7, and T8 channels have been shown to be activated in the brain. An iterative methodology was used to analyze nonlinear and nonstationary time series, i.e., the Empirical Mode Decomposition (EMD) technique for the division of time-series signals into separate components referred to as Intrinsic Mode Functions (IMFs). The validity of the method was proven on three different datasets, DEAP, DREAMER, and AMIGOS, using the Linear Support Vector Classifier (LSVC) classifier. In addition to [26], the EMD technique was also used in [29], but with a multivariate extension (MEMD) for emotion recognition. It has been shown that with various time and frequency domain features on 18 channels and Artificial Neural Networks (ANN), positive results can be obtained.

It is common to use a fusion of EEG signals with other physiological signals as has been performed by, for instance, Menon et al. [27], who calculated various features from the time and frequency domains for each signal separately EEG, ECG, and Galvanic Skin Response (GSR). The signals were preprocessed and mapped to hyperdimensional (HD) space and inserted and fused into the spatial encoder to finally obtain a single feature channel vector set representing information from all the channels. This method, based on the efficient brain-inspired Hyperdimensional computing (HDC) early fusion paradigm, has yielded promising results. Another example of using multiple datasets for the model validation process was performed in [28]. The authors provided a method based on Flexible Analytical Wavelet Transform (FAWT) that decomposes the EEG signal into different sub-band signals. They used twelve channels on the SEED dataset and signals from six channels on the DEAP dataset. Information potential (IP) was used to extract the features from the decomposed subband. The random forest classifier on the SEED dataset yielded results that outperformed the results on the DEAP dataset.

There were several studies that took advantage of the feature and channel weights acquired through the application of the ReliefF algorithm based on the DEAP dataset. For instance, the research in [30] used Principal Component Analysis (PCA) to find the main directions of the EEG features mapped to the low-dimensional space. The research performed in [19,31,32] applied a ReliefF-based channel selection algorithm to reduce the number of used channels for convenience in practical usage. Using the Probabilistic Neural Network (PNN) as a classifier, the results show that high accuracy for valence and arousal can be obtained with nine channels. To obtain approximately the same results with the SVM classifier, 19 channels for valence and 14 for arousal had to be used.

Numerous papers investigated the optimal selection process based on the SEED dataset. The authors of [33] proposed a channel selection method through weighted distributions instead of statistical parameters. Using Differential Entropy (DE) calculated from an EEG signal of only 12 electrodes, they demonstrated its superior performance over the original full pool of electrodes (62). Four different profiles (four, six, nine, and twelve channels) of the selected electrode placements according to the features of high peaks in the weight distribution and asymmetric properties in emotion processing are selected. For instance, the four electrodes selected are FT7, FT8, T7, and T8, and the other three profiles expand these for channels. The best result on the SEED dataset using the Linear Discriminant Analysis (LDA) classifier with the selected 15 channels was achieved by Pane et al. [34]. They also, as [33], calculated the DE over five subbands. The selection criteria in the Stepwise Discriminant Analysis (SDA) statistics tool were based on Wilks’ Lambda score to find the optimal channel. They then conducted several scenarios from the different number of selected channels in experiments, such as three, four, seven, and fifteen channels that were differently selected depending on which subband the DE was calculated. Transfer learning with very-deep convolutional neural networks was applied to the SEED dataset in [35] to obtain the ten best electrode positions for emotion recognition. The research performed in [36] proposed Group Sparse Canonical Correlation Analysis (GSCCA) for simultaneous EEG channel selection and concluded that both the lateral and frontal brain areas are important areas of emotional activities.

The Neighborhood Component Analysis has been used in several research papers [37,38,39,40,41] for the selection of the most significant features and the reduction in the dimensionality of the feature vectors. In particular, [37] chose the most discriminative features by using NCA weights to classify emotions into a discrete model in the Speech Emotion Recognition (SER) process, and [38] used an iterative NCA for SER to select the features prior to SVM classification into a discrete model. The paper [39] applied NCA to identify the optimal feature set from time, frequency, and statistical domain-based features for the computer-automated detection and classification of focal and non-focal epileptic seizures, [40] studies recognition of emotions from ECG signals, [41] applied NCA with a modified regularization parameter to enhance the classification performance of motor imagery tasks. To the best of our knowledge, so far, there are no published studies with the NCA for selecting the most optimal channels in recognizing emotions, so we used it for that purpose.

It is confirmed by many gender-related studies that men and women differ in emotional processes; that is, they perceive emotional stimuli in different ways. In the amygdala is an integration of sensory information that is given the appropriate emotional importance and context, and electrical stimulation results in the experience of positive or negative emotions. Study [42] researched gender differences in the neural basis of emotional memories, and [43] investigated gender-related influences on the neurobiology of emotionally influenced memory. Both studies concluded that the right amygdala in the brain is more active in women than in men in processing emotional stimuli. These studies are not directly related to the recognition of emotions based on gender but show differences in the experience of emotions in women and men. Furthermore, women show a greater physiological response than men to emotional stimuli [44,45,46].

There are many differences in emotional processing between genders, and therefore it is important that the number of respondents by gender in the dataset is balanced. However, many studies use an unbalanced number of respondents when creating a dataset. Generally, there are more men (68%) in the sample than women (32%) [47]. DREAMER with fourteen males and nine females, and AMIGOS, which consists of 17 males and 12 females, belong to this group. On the other hand, DEAP and SEED datasets recorded data on 17 men and 15 women, and seven men and eight women, respectively. From the data sets used in this paper, it is evident that the number of participants is different. Some studies have conducted research on only a single gender. For example, the authors in [48] conducted research on female emotions during motherhood and parental status, and [49] conducted a study on the development of emotional intelligence in men.

There is a distinction between research that recognizes gender based on EEG and other physiological signals and research that recognizes emotions from EEG signals depending on gender. The first approach was, for example, discussed in [50,51,52]. In particular, the study [50] showed that theta and gamma bands contain more discriminate gender information, research conducted in [51] showed that by using four entropy measurements of EEG signals, gender could be classified with very high accuracy, and [52] used deep convolutional neural networks and concluded that the main distinctive attribute was the fast beta subband. The second approach recognizes emotions from the EEG signals depending on gender. It was, for example, shown by [53] that Speech Emotion Recognition, i.e., a two-level hierarchical system, consists of a gender recognition module aimed at recognizing the speaker’s gender from the audio file and an SER module to decode the emotional state of the speaker. To the best of our knowledge, there is no scientific research in the available literature on EEG-based emotion recognition that is gender-specific on the four datasets utilized in this work.

## 3. Materials

### 3.1. Selected Datasets

Datasets are important in the process of recognizing emotions because they give researchers the opportunity to verify their models on a various number of participants of different gender, age, and culture. The datasets contain signals recorded by different measuring devices, which differ in the number of electrodes, sampling frequency, etc. In the last ten years, a large number of datasets have been presented. Four publicly available datasets have been used in this paper, namely DEAP [54], DREAMER [55], AMIGOS [56], and SEED [33]. The datasets were used to evaluate the proposed emotion recognition model, and their comparison is given in Table 1. EEG signals were collected from participants with different measuring devices that have a different number of electrodes placed according to the standard international system 10–20 [7]. Because the size of the heads may vary per participant, the distances between the positions of the electrodes are given in percentages, specifically 10% and 20%.

This paper investigates devices that have 14, 32, and 62 electrodes. It can be seen from the table below that the datasets differ in almost all parameters and, accordingly, the number of features will be different for each of them.

**Figure 1:**
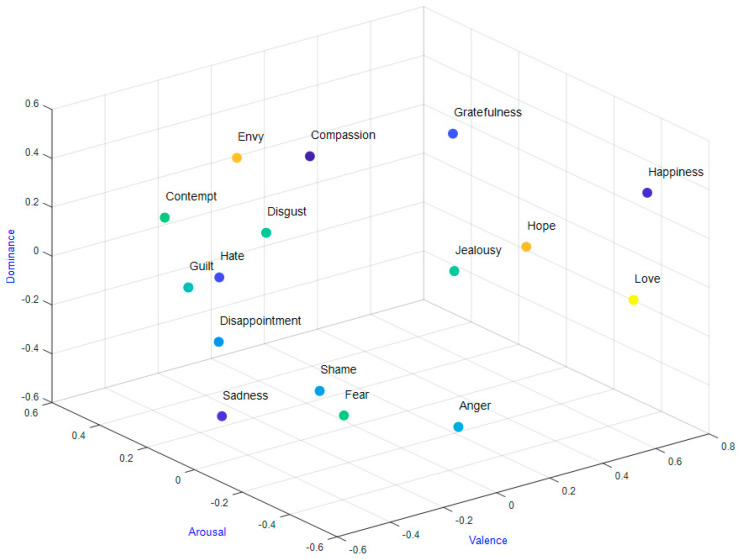
3D emotional space: Valence–arousal–dominance.

A three-dimensional model of emotions was used, as shown in Figure 1 [57]. The three dimensions are valence (ranged from sad to joyful), arousal (ranged from calm to excited), and dominance (submissive to empowered). The trials have a binary classification, i.e., they can be either high or low. The limit is set to 4.5 for the DEAP and AMIGOS datasets since the range of the rating scale is from 1 to 9. For the DREAMER dataset, the limit is 2.5 because the range rating scales from 1 to 5. EEG signals are resampled to 128 Hz regardless of the sampling frequency in order to ensure the data between datasets remain comparable.

The DEAP (Dataset for Emotion Analysis using Physiological signals) dataset was introduced in 2012. In total, 32 people aged between 19 and 37 (mean age 26.9) participated in its creation, i.e., 17 men and 15 women recorded EEG, electromyography, electrooculogram (EOG), and pulse blood volume signals while watching 40 one-minute videos. The BioSemi ActiveTwo measuring device was used to record EEG signals on which the three-second pre-trial baseline was removed. The blind source separation technique was applied to remove the EOG artifacts. In this study, we used male participants (i.e., 1, 5–7, 12, 16–21, 23, 26–30), and female participants (i.e., 2–4, 8–11, 13–15, 22, 24, 25, 31, 32) in gender-specific emotion recognition experiments. Participants 27 and 32 were removed from the analysis because they only demonstrated high dominance.

The EEG and ECG signals collected from 23 participants are an integral part of the multimodal DREAMER dataset. Fourteen male (1, 3, 6, 8, 10–15, 19, 21–23) and nine female (2, 4, 5, 7, 9, 16–18, 20) participants, aged between 22 and 33 years, watched each of the 18 emotional films clips and assessed self-emotion with the self-assessment manikins (SAM) [58] in the valence, arousal, and dominance space. EEG signals were recorded with the Emotiv EPOC wireless headset with a sampling rate of 128 Hz based on 14 electrodes. When dominance was calculated, participants 6 and 15 were excluded because both participants did not have a low dominance value.

During the construction of the AMIGOS (A dataset for Mood, personality, and affect research on Individuals and GrOupS) dataset, two experiments were conducted in which EEG, ECG, and GSR signals were recorded. In the first experiment, which is also used in this paper, 40 participants aged between 21 and 40 (mean age 28.3) watched 16 clips lasting less than 250 s. The second experiment investigated the impact of the group watching long emotional videos. Participants 9, 12, 21–24, 29, and 33 had missing data in their trials [59], and according to [60], the participants 5, 11, 28, and 30 are missing high or low valence and/or arousal, so we did not use them in our study. In addition, this research also excluded data from participants 10 and 11 because these two participants only showed high dominance values. After watching each video, the participants performed a self-assessment in seven basic emotions (neutral, disgust, happiness, surprise, anger, fear, and sadness) and in the space of valence, arousal, dominance, familiarity, and liking. Seventeen male 4, 6, 13–15, 17, 19, 20, 25–27, 29, 31, 37–40, and twelve female participants 1–3, 7, 8, 10, 16, 18, 32, 34–36 were used for gender-specific emotion recognition.

The SJTU emotion EEG Dataset (SEED) contains EEG signals recorded by the ESI Neuro Scan System measuring device from 15 participants aged 23.27 ± 2.37 (mean ± std)). Each participant conducted the experiment three times to achieve better reliability. EEG signals, lasting approximately four minutes, were collected on seven men (1, 4–6, 9, 12, 14) and eight women (2, 3, 7, 8, 10, 11, 13, 15) using 62 electrodes while participants watched Chinese film clips. Each of the three emotional categories: negative, positive, and neutral, appears in five corresponding movie clips. In this paper, only positive and negative trials from participants that can be mapped as high and low valence affective states were used, as the study authors used in [60,61,62,63,64].

### 3.2. Selected Features

To achieve the best possible information on the complexity, energy, power, standard deviation, and irregularity of the signal, various authors use signals from the time-frequency (e.g., Discrete Wavelet Transform, entropy) or spatial domain (e.g., Differential Asymmetry, Rational Asymmetry). This research applies six features from the time domain to describe the EEG signal: fractal dimension, Hjorth activity, mobility and complexity, peak-to-peak, the root-mean-square, and three from the frequency domain: band power, the differential entropy, and the power spectral density. This research uses a different set of features from different domains compared to related studies in this area. The features used in this paper provide state-of-the-art accuracy in the models where they have been used. We briefly present the nine selected features since we made a detailed analysis in a previous study [60].

The sum of the squares of the time domain samples divided by the signal length is a common feature that represents the band power [65,66,67]. A measure of signal complexity that is related to minimum description length is called differential entropy [68,69,70]. Its equation for the Gaussian distribution can be formulated as (1).

(1) $$h\left(X\right)=-\overset{+\infty }{\underset{-\infty }{\int }}\frac{1}{\sqrt{2\pi \sigma^{2}}}exp\frac{{\left(x-\mu \right)}^{2}}{2\sigma^{2}}\log\frac{1}{\sqrt{2\pi \sigma^{2}}}exp\frac{{\left(x-\mu \right)}^{2}}{2\sigma^{2}}dx=\frac{1}{2}\log2\pi e\sigma^{2}$$

where *X* follows the Gaussian distribution $N\left(\mu ,\;\sigma^{2}\right)$, *x* is a variable, and *π* and *e* are constants.

A measure of the signal complexity and irregularity can be expressed by a fractal dimension, and a fractal Brownian motion [71], Minkowski–Bouligand (box-counting dimension) [72], or the Higuchi algorithm [73], which was shown to generate optimal results [72] and is also applied in this research. In order to compute the fractal dimension with the Higuchi algorithm, let *X* (*1*), *X* (*2*), …, *X* (*N*) be a finite set of time series samples. The newly constructed time series is then defined as shown in (2) and (3).

(2) $$X_{k}^{m}:X\left(m\right),X\left(m+k\right),\ldots ,\left(m+\left[\frac{N-m}{k}\right]\cdot k\right)$$

(3) (m=1, 2,…,k)

where *m* is the initial time and *k* is the interval time.

*k* sets of *L_m_*(*k*) are calculated by (4):

(4) $$L_{m}\left(k\right)=\frac{\{\left(\sum_{i=1}^{\left[\frac{N-m}{k}\right]}\left|X\left(m+ik\right)-X\left(m+\left(i-1\right)\cdot k\right)\right|\right)\frac{N-1}{\left[\frac{N-m}{k}\right]\cdot k}\}}{k}$$

where 〈L(k)〉 denotes the average value of *L_m_*(*k*), and a relationship exists as follows in (5):

(5) $$\langle L\left(k\right)\rangle \propto k^{-D}$$

Moreover, the fractal dimension can be obtained by logarithmic plotting between different *k* and its associated 〈L(k)〉. Hjorth parameters [74] for a signal *x* of length *N* are the commonly used features in signal processing [75,76]. Hjorth Activity represents the squared standard deviation of the amplitude (mean power of the signal), which is shown in (6):

(6) $$Activity\left(x\right)=\frac{\sum_{n=1}^{N}{\left(x\left(n\right)-\bar{x}\right)}^{2}}{N}$$

where $\bar{x}$ stands for the mean of *x*. Hjorth Mobility measures a standard deviation of the slope with reference to the standard deviation of the amplitude (mean frequency of the signal) and can be calculated by (7):

(7) $$Mobility\left(x\right)=\sqrt{\frac{var\left(x^{\prime }\right)}{var\left(x\right)}}$$

where *x’* denotes the derivate of signal *x*. The third parameter, Hjorth Complexity, is the number of standard slopes, i.e., the measure of the deviation of the signal from the sine shape (8):

(8) $$Complexity\left(x\right)=\frac{Mobility\left(x^{\prime }\right)}{Mobility\left(x\right)}$$

The Root-Mean-Square [77,78,79] and the Peak-to-Peak [65,80] are ordinary methods that are frequently used for measuring the amplitude of the signal. The latter feature represents the difference between the maximum and minimum values in signal *x*. A well-known method for calculating average energy from different frequency bands is Power Spectral Density [81,82,83,84]. This research uses the mean value of Welch’s method.

## 4. Methodology

This chapter describes the methodology used in the emotion recognition process. The first subchapter describes how two-dimensional holographic feature maps are created from the values of the signal characteristics represented in space. ReliefF and NCA methods for automatic selection of EEG channels containing the most relevant information about emotions are then described. Finally, it is described how a model was constructed consisting of modules for extracting and merging features and classification into a three-dimensional emotional space.

As there is still no model that will be maximally reliable given the person and the environment in which the person is at the time of reading the EEG signal, it is necessary to make additional efforts to improve these models. The nature of the EEG signal, which has a large amount of noise, makes it difficult for researchers to construct the optimal and efficient model of emotion recognition. Discrete wavelet transformation (DWT) decomposed signals into delta, theta, alpha, beta, and gamma subbands, and the characteristics of the signal in the time and frequency domain are calculated from them. In this study, we used our model to recognize emotions by using the advantages of holographic feature maps (HOLO-FM) that can be used to represent spatial and spectral information of the signal. With our holographic feature maps, we aimed to give a new perspective to this research area. We used them because of their proven efficacy compared to studies that have reached the state-of-the-art level of accuracy.

In order to optimize the model and improve the accuracy of the classification, this paper focuses on investigating the most relevant channels for efficient EEG-based emotion recognition. This paper suggests using ReliefF and Neighborhood Component Analysis methods to deal with the aforementioned challenges. Holographic feature maps constructed with critical channels that were selected by means of the ReliefF method are defined as R-HOLO-FM. The feature maps based on the NCA methodology are defined as N-HOLO-FM. The analysis of the effectiveness of the R-HOLO-FM method is extended to the study of men and women in the three-dimensional space of valence, arousal, and dominance.

### 4.1. Feature Maps Creation

Gabor [85] invented holography in 1948, which is a method of capturing and reproducing a three-dimensional image of an object using light interference and diffraction with the help of coherent light without the use of optical lenses [60]. The hologram is created by recording the interference fringes between the reference wave coming from a coherent light source (laser) and the wave diffracting from the object and going to the recording medium, where it interferes with the reference wave. Complete spatial information about the object is contained in the amplitude and phase of the object wave. Therefore, this research also applies the same principle to map a three-dimensional object into the plane and show it as a two-dimensional image [86]. This is performed with Computer-Generated Holography, which is a technology suitable for generating holograms from a synthetic three-dimensional graphic model [87].

**Figure 2:**
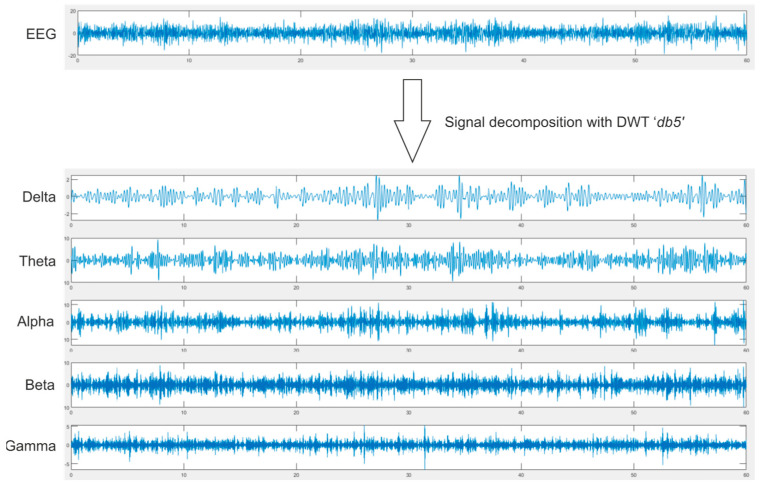
Decomposition of the EEG signal in sub bands with Discrete Wavelet Transform using ‘db5′ mother wavelet.

The HOLO-FM method is based on the creation of two-dimensional feature maps from the spatial characteristics of the signals by using computer-generated holography. As shown in Figure 2, the EEG signal was divided into delta (0–4 Hz), theta (4–8 Hz), alpha (8–16 Hz), beta (16–32 Hz), and gamma (32–64 Hz) subbands using a ‘db5′ mother wavelet of the DWT. Nine signal characteristics are then calculated for each subband, and the position of the point in the 3D space is defined by displaying this value at the electrode location, which is defined by a standard international 10–20 system. Thus, nine feature maps are obtained for each subband, and 45 for each EEG signal.

**Figure 3:**
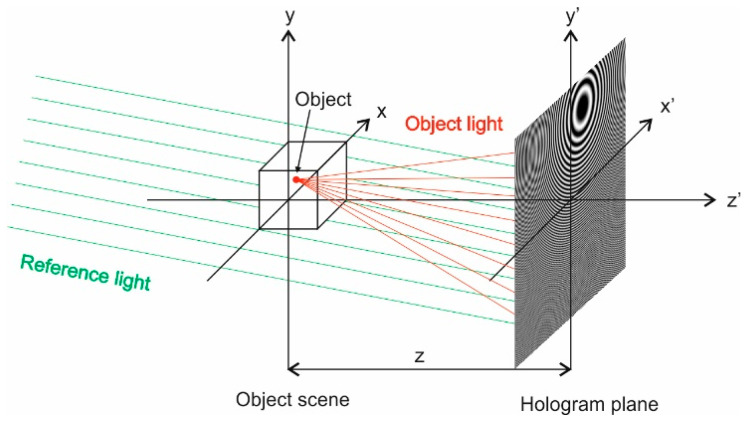
The construction of holographic feature maps using Computer-Generated Holography.

Figure 3 [60] shows the HOLO-FM method, i.e., the construction of holographic feature maps using Computer-Generated Holography. Unlike a photograph that can record the distribution and intensity of light waves, a hologram can additionally record their direction and phase and is able to distinguish between “very distant light” and “dimmed near light”, thus achieving a three-dimensional effect. We used a CGH algorithm [86] to create an off-axis hologram to obtain a two-dimensional feature map. The scene of the object is rotated in such a way that the illumination comes along the Z-axis. Each individual point in space represents the value of the signal characteristic. The classical hologram is calculated for each individual point in space, and the procedure is repeated for all points of all electrodes.

**Figure 4:**
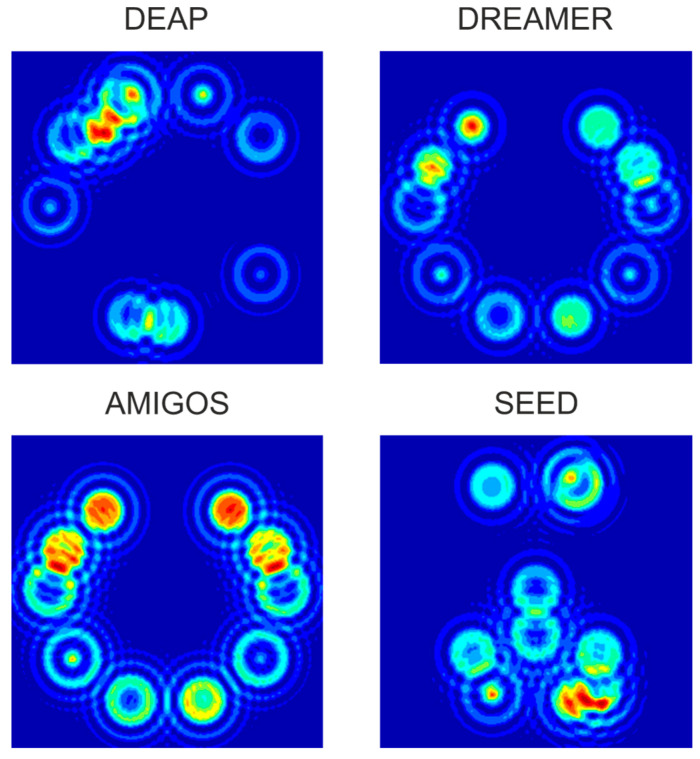
Example of holographic feature maps for each dataset.

Figure 4 shows an N-HOLO-FM example for the first participant of all datasets. Ten electrodes selected by the NCA method are shown for Differential Entropy (DE) signal characteristics on the gamma subband. The red color represents active electrodes, while the dark blue color denotes that the electrode is not active at all.

### 4.2. Channel Selection

The electrodes are attached to the scalp to obtain brain signals. The number of electrodes varies from 1 to 256 for different EEG headsets. The international 10–20 system indicates the position of the electrodes on the human head in such a way that the letters F, P, T, and O denote the frontal, parietal, temporal, and occipital lobes. The letter C does not represent a lobe but is used to identify the central part of the head, A represents the position of the front electrode, while the letter Z (zero) is used to indicate the position of the electrode on the midline between the two hemispheres of the brain. The electrodes in the left hemisphere are denoted by odd numbers 1, 3, 5, and 7, while the positions of the electrodes in the right hemisphere are denoted by even numbers 2, 4, 6, and 8. The most commonly used electrodes, according to the research conducted in [47], are presented in Table 2. This shows that most researchers are of the view that the frontal lobe plays an important role in the emotion recognition process.

Considering the number of electrodes, the level of comfort of wearing the EEG measurement device, the time required to mount the device on the subject’s head, the complexity and processing time of all recorded signals, it is reasonable to use as few electrodes as possible to solve these problems, and that is a major task of the EEG channel selection process. We conducted additional research, and through the procedure, we reached the optimal number of the ten most relevant electrodes using the ReliefF and NCA methods which are briefly described below.

With the aim to select the most optimal channels, we used the ReliefF method proposed by Kononenko [88] because it showed its effectiveness in the works [19,30,31]. The ReliefF algorithm is a widely used feature selection method that has a lot of advantages, such as simple principles and fast computing speed. It is used to compute the weights of features based on sample learning. The basic idea of the algorithm is to determine the weight of features, i.e., to assess the quality of the features according to their ability to distinguish between samples that are close to each other. The assessment of feature quality is determined by the ability of the nearest neighbor of one class to distinguish the nearest neighbors of any two classes. Channel selection can be performed based on the feature selection results. The Algorithm 1 is represented by pseudocode [89]:
**Algorithm 1.** Pseudocode of ReliefF algorithm:Inputs: Instance set S and the number of classes C Output: Weight vector w Step 1: For any feature f_a_, *a* = 1, 2, …, *d*, set the initial weight *w_a_* = 0 Step 2: for *i* = 1 to *m* do Randomly select *x_i_* from *S*; Select the *k*-nearest neighbors *h_j_* from the same class of *x*; Select the *k*-nearest neighbors *m_j_*(*c*) from different class from *x* for *a* = 1 to *d* do Update the weight by (9):
(9)$$w\left(a\right)∶=\frac{w\left(a\right)-\sum_{j=1}^{k}\frac{dist\left(a,x_{i},h_{j}\right)}{m-k}+\sum_{c\neq class\left(x_{i}\right)}\left\{\frac{P\left(c\right)}{1-P\left[class\left(x_{i}\right)\right]}\times \sum_{j=1}^{k}dist\left[a,x_{i},m_{j}\left(c\right)\right]\right\}}{\left(m-k\right)}$$ End End  
where *dist*(*a*, *x*, *y*) is the distance between instances *x* and *y* under the feature *a*, *P*(*c*) denotes the probability of the *c*-th class which can be obtained as the ratio between the size of the *c*-th class and the total number of instances, *m_j_*(*c*) denotes the *j*-th sample from the *c*-th class, *m* is the number of iterations, and *k* is the number of nearest neighbors.

Features can be ranked by their weight. A high weight means that the feature is important for distinguishing samples and vice versa. Firstly, N features were selected according to the rank of each feature. Secondly, the channels that contain those features were selected.

Goldberger et al. [90] proposed Neighborhood Component Analysis, a distance-based method that uses the Mahalanobis distance in the kNN classification algorithm. The idea is that with a certain probability *p_ij_* each point *i* selects its neighbor point *j* and inherits its class label (10):

(10) $$p_{ij}=\frac{exp\left(-\|Ax_{i}-Ax_{j}\|{}^{2}\right)}{\sum_{k\neq i}exp\left(-\|Ax_{i}-Ax_{k}\|{}^{2}\right)},\;p_{ii}=0$$

with objective function defined as (11)

(11) $$f\left(A\right)=\underset{i}{\sum }\underset{j\in C_{i}}{\sum }p_{ij}=\underset{i}{\sum }p_{i}$$

where *A* is the transformation matrix, *x_i_* are real-valued input vectors in *R^D^*, and *C_i_* denotes the corresponding class labels *c*_1_, …, *c_n_*.

With this scheme, the NCA is able to extract data that contains useful information and consequently reduce the dimensionality of the data. The NCA is an effective feature selection model that generates and selects positively weighted features. According to the authors of the study [39], they were the first to use the NCA algorithm for the selection of significant features for EEG signals.

**Figure 5:**
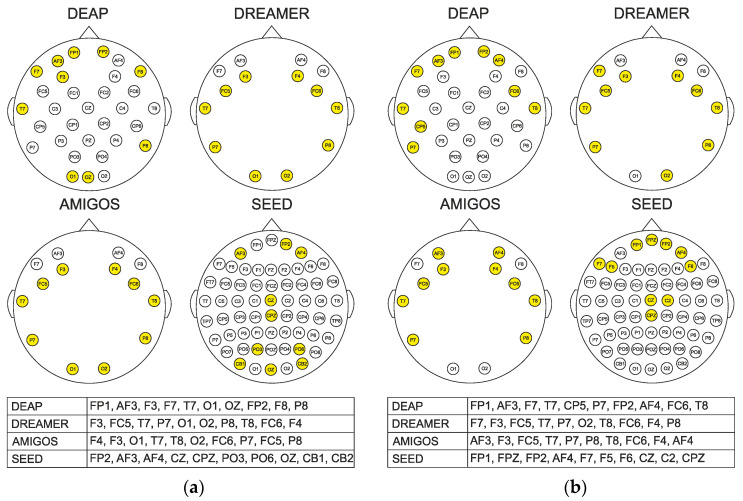
Head map with top 10 channels highlighted in yellow that are selected with (**a**) ReliefF and (**b**) NCA for each dataset.

**Figure 6:**
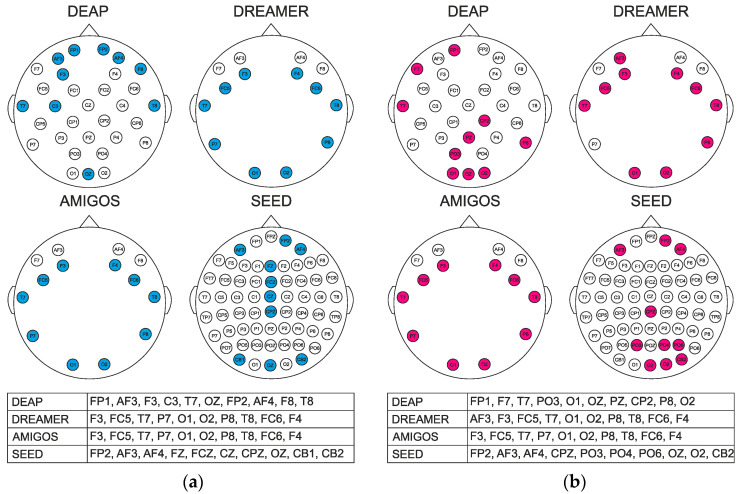
Head map with top 10 channels selected with ReliefF on (**a**) male (highlighted in blue) and (**b**) female (highlighted in red) participants for each dataset.

In this study, the selection of the most significant EEG channels is aimed at improving the accuracy of the classification. The weights are obtained with the proposed ReliefF and NCA algorithms, and the most important channels are selected based on the highest weights. The selection procedure was performed in such a way that for each trial, nine signal characteristics were calculated on each individual sub-band for all channels. Recall that DEAP has 32, DREAMER and AMIGOS 14 each, and SEED 62 channels. The channels were sorted by weight. The top ten EEG channels that best reflect emotional characteristics were selected. For the SEED dataset, we calculated the holographic feature maps separately for each experiment with the ten most relevant channels previously selected. The results are denoted in the percentage of accuracy and serve as the mean of all three experiments. Figure 5 and Figure 6 show the most relevant channels for SEED from all three experiments. The top ten channels were selected with the same procedure as for the other datasets. Since ReliefF and NCA are different methods, we wanted to investigate whether we would find a different set of channels. Thus, although the electrodes in Figure 5 are not identical for both methods, it is observed that the frontal electrodes dominate. That is, both methods emphasized the importance of the frontal lobe in the process of recognizing emotions.

The channels selected by ReliefF and NCA methods on all datasets are consistent with the conclusion given in [26], i.e., the most active areas of the brain during emotion processing are the frontal, parietal, and temporal areas. Moreover, the survey study [47] presented the percentage of use of certain channels in numerous previous papers, and the data are also in line with our proposal.

### 4.3. Model Construction

**Figure 7:**
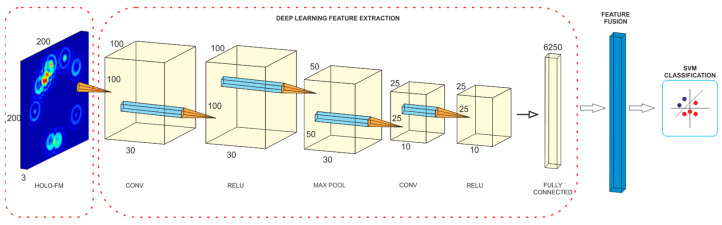
Flow chart of feature extraction, fusion, and classification.

A Convolutional Neural Network to extract features from the holographic feature maps was constructed for each signal characteristic (in total, nine are used in this study). The features extracted from each individual CNN are merged into a feature matrix from which the machine-learning algorithm derives a classification of user emotional states. The three-component model is shown in Figure 7. CNN is a feedforward network that is often used for learning and extracting features but also for classification. Because deep-learning-based methods can learn highly representative features [91], we used a deep CNN to extract features from the input images that represent the holographic feature maps. We used a Convolutional Neural Network consisting of two two-dimensional convolutional layers, two activating Rectified Linear Unit (ReLU) layers, a max-pooling layer, and, finally, a fully connected layer that was used to extract more significant features. The dimensions of the color images used as input to the CNN are 200 × 200 × 3. The 2D convolutional kernel is 2 × 2 pixels in size. The stride equals two, and no padding was used. The network options are 0.001, 0.04, and 32 for initial learn rate, L2 regularization, and mini-batch size, respectively, and a Stochastic gradient descent with momentum was used for optimization.

The extracted high-level features of all nine convolutional networks are merged into a final matrix that is the input to the machine learning algorithm. The final part of the model is needed to provide accurate, predicted results for emotion recognition. The Support Vector Machine classifier with a polynomial kernel was used to distinguish high and low levels of valence, arousal, and dominance.

The holdout and the k-fold cross-validation methods are generally the criteria for evaluating the success of a model. The most commonly used method to evaluate the success of a model is k-fold cross-validation. The data set is divided into k subsets where the training set is formed from k–1 subsets, and the remaining k subset is used as a test set. Accuracy is calculated for each test set, and the procedure is repeated k times. In this work, a statistic 10-fold cross-validation technique was used to assess the accuracy of our model. The idea is to divide the input set into ten equal parts, nine of which are used for training and one for testing. The final accuracy of the model is obtained by calculating the average value for all folds, and later for all participants.

## 5. Results and Discussion

In emotion recognition, channel selection has proven to be an important factor in helping machine learning algorithms work more efficiently. Hereinafter, we present the experimental results of our model for emotion recognition based on a reduced set of channels. Table 3 gives an overview of the accuracy and F1-scores (average of F1-score for each class) for both R-HOLO-FM and N-HOLO-FM methods on all datasets in the valence, arousal, and dominance space. It can be seen that the R-HOLO-FM method outperforms N-HOLO-FM in the rate of accuracy and in F1-score results by up to four percentage points in most cases. The expected values of classifying randomly, classifying according to the majority class, and classifying by choosing a class with the probability of its occurrence in the training data are given as baseline values. The best classifications rates and F1-scores on all datasets in all three emotion dimensions are highlighted in bold. Using an independent one-sample *t*-test (*p* < 0.01), it was obtained that all classification F1-scores are significantly better than the class ratio baseline level. The results of the R-HOLO-FM and N-HOLO-FM methods are not significantly different for any of the three affective states according to a related two-sided *t*-test (*p* < 0.05).

Table 4, Table 5, Table 6 and Table 7 provide an overview of the accuracy of both R-HOLO-FM and N-HOLO-FM methods on all datasets in the valence, arousal, and dominance space. The best accuracy score in each table is highlighted in bold. In our experiments, we used the top ten channels selected by ReliefF and NCA methods, and recall that SEED has 62, DEAP 32, and DREAMER and AMIGOS have 14 electrodes on their measuring devices. Thus, for DEAP, we used 31.25% of the most optimal channels, for DREAMER and AMIGOS 71.43%, while for SEED, we used only 16.13% of all available channels. R-HOLO-FM and N-HOLO-FM methods achieved up to a seven percentage points better level of accuracy than our previous work [60], which shows that we successfully improved the model performance with ReliefF and NCA methods of selecting EEG channels containing the most relevant information during the emotion recognition process. The comparison results for each dataset are given in Table 4, Table 5, Table 6 and Table 7. Abbreviations V, A, and D are used in the tables to denote valence, arousal, and dominance. Apart from the basic ones, which are actually the studies of the authors of the data sets, we chose studies where the authors used a reduced set of EEG channels, whether the channels were selected manually or selected by some automatic method, and which classify human emotions in the dimensional space.

The results obtained by R-HOLO-FM and N-HOLO-FM methods with comparable studies are given in Table 4. Out of a total of 12 studies, only [24,26] did not present results in the arousal dimension, but none investigated the dominance dimension as is conducted in this paper. The number of selected channels varies from paper to paper, so the least, only five, were selected in [24], while [21,29] used 18 different channels, and the authors of the DEAP dataset used all channels. Channels were mostly selected manually based on the experience of other authors in [21,22,23,24,26,28,29], while the ReliefF method was used in [32], and Wang et al. used the NMI method for selecting the most optimal channels [25].

The highest classification accuracy using ten channels from the frontal lobe of 91.10% for valences and 91.30% for arousal was achieved in [22]. However, the results are not shown over the whole EEG signal but only over subbands, so this result refers to the beta subband. The approach of [23] proposed a similar method with the same set of channels as [22], but using kNN classifiers, they achieved a worse accuracy rate by about five percentage points for valence and about percentage points for arousal. Using 18 channels, which is more than half of the total number of the DEAP dataset, studies [21] as well as [29] conducted research, and in comparison, they achieved results of 85.74% and 72.87% for valence, while in arousal dimension they achieved 87.90% and 75%. The difference in classification accuracy greater than 10 percentage points is significant, and it is obvious that the proposed MEMD method with ANN classifier needs to be improved to achieve the accuracy rate method used by Li. In addition to the aforementioned [21] undertook additional research with 10 and 14 channels. The results obtained over all frequency bands of each channel combination are 82.48% and 84.53% in the valence space and 83.27% and 85.26%, in the arousal space for 10 and 14 channels.

Wang [25] and Zhang [32] used the Normalized Mutual Information and ReliefF methods for channel selection, respectively, and were the only ones to suggest the use of different numbers of channels for both dimensions in 2D emotional space. An accuracy of 74.41% for valence with eight channels and 73.64% for arousal with 10 channels were obtained with support vector machines by Wang. On the other hand, Zhang used the probabilistic neural network (PNN) as a classifier and showed that with nine channels, an accuracy of 81.21% for valences and with eight channels, an accuracy of 81.76% for arousal could be obtained. The same number of channels as in [25], in the study of emotion recognition, was used by Msonda [26], who showed that the frontal, parietal, and temporal are common brain areas that are active during emotion processing. The authors found an accuracy rate of 67.00%, which is better by about 10% than the results published by the authors of the DEAP dataset [54] but still worse than all other comparable studies presented in this paper. The result suggests that using the Empirical Mode Decomposition technique to split the time series signal into Intrinsic Mode Functions will not yield promising results. However, on DREAMER and AMIGOS datasets, on which the authors also conducted research, they achieved a classification accuracy of 78.00% and 80.00%, respectively, which consequently means that it makes sense to further optimize the proposed method.

The smallest number of channels is used by studies [24,28]. Özerdem selected five channels: P3, FC2, AF3, O1, and Fp1, which mostly coincide with the selected channels in our study. By utilizing the MultiLayer Perceptron Neural Network (MLPNN), they obtained a score of 77.14% for positive and negative emotions and showed that this method was more successful than SVM, which gave them an accuracy of 72.92%. With one channel more than in the previously mentioned method (T7, T8, CP5, CP6, P7, and P8), the authors in [28] reported 79.99% for valence and 79.95% for arousal, which is fairly effective for emotion recognition.

DREAMER and AMIGOS datasets were published not so long ago, so there are only a few comparative papers in addition to the basic ones. As in the DEAP data set experiment, Msonda [26] used a Linear Support Vector Classifier and achieved a score of 80.00%, which is significantly more than Katsigiannis showed. The authors of the DREAMER dataset [55] have shown classification accuracy in all three dimensions, but both our R-HOLO-FM and N-HOLO-FM methods outperform them with scores of 90.76%, 92.92%, and 92.97% for valence, arousal, and dominance, respectively. In addition, the proposed methods show that the model of [60] is further optimized by a few percentage points. Using spectral power features, authors of the AMIGOS dataset [56] have reached the emotion prediction of 57.60% for valence and 59.20% for arousal. It can be seen that they underperform the accuracy of results for both affective states in comparison with all the other studies presented in Table 6.

Using two datasets as a basis for model verification, the authors in [27] fused preprocessed EEG, GSR, and ECG signals, previously mapped into hyperdimensional space. The brain-inspired hyperdimensional computing paradigm yielded results on the DEAP dataset of 76.70% and 74.20% in the valence and arousal dimensions, respectively. The obtained accuracy rate still underperforms both of our methods. From the AMIGOS point of view, the valence of 87.10% is approximately the same as in [60] and slightly worse than the R-HOLO-FM and N-HOLO-FM methods. However, the arousal value is about ten percentage points higher in both previously mentioned methods.

On the SEED set that originally contained signals from 62 EEG channels, the authors mostly used a reduced set of 10 to 15 channels. The SEED dataset authors [33] achieved 86.08% for emotion recognition with all channels, but in the same paper, they conducted research for four, six, nine, and twelve channels and obtained an accuracy rate for the valence of 82.88%, 85.03%, 84.02%, 86.65%, respectively. By reducing 50 EEG channels, the authors showed its superior performance with an accuracy of 86.65% over the original full pool of electrodes. R-HOLO-FM and N-HOLO-FM methods outperformed their approach, as well as the method proposed in [36], with a classification accuracy of 88.19% and 88.31%, respectively. It is worth noting that the Group Sparse Canonical Correlation Analysis method for simultaneous EEG channel selection, proposed by Zheng, obtained a result of 83.72% for the 12 most optimal channels. The same number of the most important channels from the frontal, parietal and central lobes were used by Gupta [28], who, in addition to the DEAP dataset, conducted the emotion recognition survey on the SEED dataset and achieved a very good result of 90.48% with the Random Forest classifier.

The highest classification accuracy achieved in [34] is 99.85%. Authors, such as Zheng [33], tested several scenarios on their model with a different number of channels: three, four, seven, but with 15 channels, they achieved the mentioned result. Finally, the approach of [35] proposed a method that utilizes the outermost-10 channels with which they obtained the best mean accuracy of 91.67%. In their interesting study, they obtained 83.83% with modified very-deep convolutional neural networks using the outer-10 channels, 70.60% with inner-10, and 54.75% with innermost-10 channels.

This study based on holographic feature maps, which selected the most optimal channels, has demonstrated that R-HOLO-FM and N-HOLO-FM are effective methods for emotion recognition with a reduced set of channels that perform better than most comparable approaches previously published. In the DEAP, DREAMER, and AMIGOS datasets, valence, arousal, and dominance emotion states are divided into low and high class. In the SEED dataset, the emotional states are divided into positive and negative values, corresponding to valence. By analyzing the available literature, we were unable to find models that use a reduced set of channels to classify emotions into 3D emotional space, except for DREAMER data set authors who use all measuring device channels. As determining dominance is also very important in recognizing emotions, more research is needed in this direction.

Men and women have different perceptions of emotional stimuli, as evidenced by numerous studies investigating gender differences. Therefore, we also expanded our research and conducted experiments on how gender affects the recognition of emotions using different datasets. Since we have achieved better results on all bases in previous research using the R-HOLO-FM method, we used it to recognize the emotions of women and men. Given that slightly less than 70% of studies use an unbalanced set in the sense that men predominate, and as it is known that men and women react differently to emotional stimuli, the results of these studies would certainly be different if approximately the same number of men and women participated. DREAMER consists of fourteen males and nine females, and AMIGOS of 17 males and 12 females, making them unbalanced datasets, and the other two datasets, i.e., DEAP and SEED, have a ratio of 17 males and 15 females, respectively, seven men and eight women. For this reason, we wanted to investigate what results we will find when using the model proposed in this paper on men and then on women. The test results are available in Table 8 and Table 9.

There is an obvious gender difference for all emotional dimensions and in all datasets. Thus, the most pronounced difference in valence, which ranges from sad to joyful, on the DEAP, AMIGOS, and SEED datasets is approximately five percentage points in favor of men, while for DREAMER, the value of valence is almost the same. From the results, it can be seen that the same is true for arousal (ranging from calm to excited), where the largest difference between the genders in DEAP is approximately seven percentage points, and the smallest in AMIGOS, which is two percentage points. From the above, we can conclude that men are more likely to recognize emotions in valence and arousal affective states. Finally, the dominance level in all three datasets is higher for women than for men by an average of five percentage points, and recall that low dominance means submissive/without control, and high dominance means dominance/empowered.

In summary, we can conclude that, although there is a relatively small number of subjects in each dataset, it still includes a total of 99 respondents, of which 55 are men, and 44 are women. Although there are numerous studies on recognizing gender from physiological signals, very few deal with recognizing emotions depending on gender. Here, we presented our interesting findings, and as this is not a large set of participants, it is necessary to continue to conduct research on the matter.

## 6. Conclusions

The use of an EEG headset with a large number of channels increases the complexity of hardware and computing and, consequently, the process of recognizing the emotional state of people because part of the channel is not relevant for recognizing emotions and generates unnecessary noise. This research proposes a reduced set of channels, which improves the aforementioned challenges that scientists face today, and also raises the level of classification accuracy using the ReliefF and Neighborhood Component Analysis methods. Computer-generated holography was used to construct R-HOLO-FM and N-HOLO-FM feature maps with a reduced set of EEG channels from the characteristics of the signals displayed in three-dimensional space. The results obtained using deep learning neural networks and the classification of machine learning methods effectively improve the rate of emotional recognition. Furthermore, the R-HOLO-FM method was applied for both men and women, and the results show that gender differences are visible. The suggested approach was verified on four different datasets, i.e., DEAP, DREAMER, AMIGOS, and SEED, where EEG signals were recorded with a different number of channels. The results of this study are presented in the valence, arousal, and dominance space of emotions to assess the effectiveness of the model. Based on the results, the authors of this paper are of the opinion that the use of the proposed method can play a significant role in the process of recognizing emotions.

There is still a need for further research in this field, so in our future work, we will investigate the effectiveness of our model for cross-dataset, i.e., we will use different datasets for training and others for testing the model. Further, special efforts will be made in the research with the aim of reducing the set of signal characteristics and channels in order to improve the speed of calculations which should enable the usage of the model in real-time. Moreover, we will continue to work on recognizing emotions depending on gender because a very interesting area of research is opening up in this direction.

## Figures and Tables

**Table 1:** Comparison between datasets.

Dataset	DEAP	DREAMER	AMIGOS	SEED
Participants	32	23	40	15
Trials	40	18	16	10
Channels	32	14	14	62
Affective states	Valence Arousal Dominance	Valence Arousal Dominance	Valence Arousal Dominance	Valence
Rating scale range (threshold)	1–9 (4.5)	1–5 (2.5)	1–9 (4.5)	N/A

**Table 2:** The percentages of use of each EEG channel.

Percentage	Channels
>75%	F4, F3
60–75%	T7, FP1, FP2, T8, F7, F8
45–60%	O1, P7, P8, O2
30–45%	FC5, FC6, C4, C3, AF3, AF4
<30%	P3, P4, Pz

**Table 3:** Accuracy and F1-score for proposed and baseline methods in 3D emotional space per dataset.

			DEAP	DREAMER	AMIGOS	SEED
R-HOLO-FM	Valence	Accuracy	**83.26**	**90.76**	**88.54**	88.19
		F1-score	**87.13**	**89.09**	**89.79**	88.51
	Arousal	Accuracy	**83.85**	**92.92**	**91.51**	N/A
		F1-score	**86.80**	**89.25**	**87.77**	N/A
	Dominance	Accuracy	**88.58**	**92.97**	**90.34**	N/A
		F1-score	86.56	**89.47**	87.83	N/A
N-HOLO-FM	Valence	Accuracy	81.88	86.12	88.53	**88.31**
		F1-score	86.16	88.48	87.90	**88.59**
	Arousal	Accuracy	82.45	89.07	91.32	N/A
		F1-score	85.76	88.58	87.58	N/A
	Dominance	Accuracy	88.35	89.82	86.10	N/A
		F1-score	**89.95**	89.04	**87.89**	N/A
Random	Valence	Accuracy	51.33	48.79	49.57	43.33
		F1-score	50.42	48.21	49.43	43.21
	Arousal	Accuracy	49.06	51.45	49.78	N/A
		F1-score	48.13	49.21	46.96	N/A
	Dominance	Accuracy	50.70	51.21	57.33	N/A
		F1-score	49.35	45.87	57.33	N/A
Majority	Valence	Accuracy	63.13	61.11	56.47	50.00
		F1-score	38.70	37.93	36.09	33.33
	Arousal	Accuracy	63.75	72.43	65.95	N/A
		F1-score	38.93	42.02	39.74	N/A
	Dominance	Accuracy	66.72	77.05	54.74	N/A
		F1-score	40.02	43.52	35.38	N/A
Class ratio	Valence	Accuracy	45.94	48.79	51.72	50.67
		F1-score	47.66	48.79	49.14	49.33
	Arousal	Accuracy	45.94	39.61	45.69	N/A
		F1-score	42.03	40.10	43.97	N/A
	Dominance	Accuracy	42.03	35.75	51.72	N/A
		F1-score	38.59	30.92	51.72	N/A

**Table 4:** Comparison with other studies with reduced channel set on DEAP dataset.

Study	Used Feature(s)	Classification Method(s)	Number of Channels	Best Accuracy
Koelstra et al. [54]	PSD	NB	32	V: 57.60 A: 62.00
Li et al. [21]	Entropy and energy	kNN	18	V: 85.74 A: 87.90
Bazgir et al. [22] *	Entropy and energy	SVM	10	**V: 91.10** **A: 91.30**
Mohammadi et al. [23]	Entropy and energy	kNN	10	V: 86.75 A: 84.05
Özerdem et al. [24]	Various time and frequency domain features	MLPNN	5	V: 77.14
Wang et al. [25]	Band energy (spectrogram)	SVM	8 for V 10 for A	V: 74.41 A: 73.64
Msonda et al. [26]	EMD IMFs	LSVC	8	V: 67.00
Menon et al. [27] **	Various time and frequency domain features	HDC	Feature channel vector set	V: 76.70 A: 74.20
Gupta et al. [28]	IP	RF	6	V: 79.99 A: 79.95
Mert et al. [29]	Various time and frequency domain features	MEMD + ANN	18	V: 72.87 A: 75.00
Zhang et al. [32]	Band power	PNN	9 for V 8 for A	V: 81.21 A: 81.76
Our method	R-HOLO-FM	CNN + SVM	10	V: 83.26 A: 83.85 D: 88.58
Our method	N-HOLO-FM	CNN + SVM	10	V: 81.88 A: 82.45 D: 88.35

V: Valence, A: Arousal, D: Dominance, PSD: Power Spectral Density, NB: Naïve Bayes, kNN: k-Nearest Neighbor, SVM: Support Vector Machine, MLPNN: MultiLayer Perceptron Neural Network, EMD: Empirical Mode Decomposition, IMF: Intrinsic Mode Function, LSVC: Linear Support Vector Classifier, HDC: Hyper-Dimensional Computing, IP: Information potential, RF: Random Forest, MEMD: Multivariate Empirical Mode Decomposition, ANN: Artificial Neural Network, PNN: Probabilistic Neural Network, CNN: Convolutional Neural Network. * The average classification accuracy on Beta band. ** GSR, ECG and EEG signals were used.

**Table 5:** Comparison with other studies with reduced channel set on DREAMER dataset.

Study	Used Feature(s)	Classification Method(s)	Number of Channels	Best Accuracy
Katsigiannis et al. [55]	PSD	SVM	14	V: 62.49 A: 62.17 D: 61.84
Msonda et al. [26]	EMD IMF	LSVC	8	V: 80.00
Our method	R-HOLO-FM	CNN + SVM	10	**V: 90.76** **A: 92.92** **D: 92.97**
Our method	N-HOLO-FM	CNN + SVM	10	V: 86.12 A: 89.07 D: 89.82

V: Valence, A: Arousal, D: Dominance, PSD: Power Spectral Density, SVM: Support Vector Machine, EMD: Empirical Mode Decomposition, IMF: Intrinsic Mode Function, LSVC: Linear Support Vector Classifier, CNN: Convolutional Neural Network.

**Table 6:** Comparison with other studies with reduced channel set on AMIGOS dataset.

Study	Used Feature(s)	Classification Method(s)	Number of Channels	Best Accuracy
Miranda et al. [56]	PSD, SPA	SVM	14	V: 57.60 A: 59.20
Msonda et al. [26]	EMD IMF	LR	8	V: 78.00
Menon et al. [27] *	Various time and frequency domain features	HDC	Feature channel vector set	V: 87.10 A: 80.50
Our method	R-HOLO-FM	CNN + SVM	10	**V: 88.54** **A: 91.51** **D: 90.34**
Our method	N-HOLO-FM	CNN + SVM	10	V: 88.53 A: 91.32 D: 86.10

V: Valence, A: Arousal, D: Dominance, PSD: Power Spectral Density, SPA: Spectral Power Asymmetry, SVM: Support Vector Machine, EMD: Empirical Mode Decomposition, IMF: Intrinsic Mode Function, LR: Linear Regression, HDC: Hyper-Dimensional Computing, CNN: Convolutional Neural Network. * GSR, ECG and EEG signals were used.

**Table 7:** Comparison with other studies with reduced channel set on SEED dataset.

Study	Used Feature(s)	Classification Method(s)	Number of Channels	Best Accuracy
Zheng et al. [33]	Feature map from DE	DBN + SVM	12	V: 86.65
Gupta et al. [28]	IP	RF	12	V: 90.48
Pane et al. [34]	DE	SDA + LDA	15	**V: 99.85**
Cheah et al. [35]	Extracted with VGG14	VGG14 1D kernel (T-then-S)	10	V: 91.67
Zheng [36]	Raw EEG features	GSCCA	12	V: 83.72
Our method	R-HOLO-FM	CNN + SVM	10	V: 88.19
Our method	N-HOLO-FM	CNN + SVM	10	V: 88.31

V: Valence, A: Arousal, D: Dominance, DE: Differential Entropy, DBN: Deep Belief Networks, SVM: Support Vector Machine, IP: Information Potential, RF: Random Forest, SDA: Stepwise Discriminant Analysis, LDA: Linear Discriminant Analysis classifier, VGG: Visual Geometry Group, GSCCA: Group Sparse Canonical Correlation Analysis, CNN: Convolutional Neural Network.

**Table 8:** Accuracy for male subjects with the channels selected with the ReliefF.

Dataset	Valence	Arousal	Dominance
DEAP	82.55	82.27	88.81
DREAMER	90.26	91.87	93.24
AMIGOS	83.63	87.84	90.40
SEED	82.07	N/A	N/A

**Table 9:** Accuracy for female subjects with the channels selected with the ReliefF.

Dataset	Valence	Arousal	Dominance
DEAP	87.82	89.26	82.12
DREAMER	89.58	93.27	89.42
AMIGOS	88.92	92.08	85.44
SEED	87.70	N/A	N/A

## Data Availability

This study utilizes the publicly available datasets from DEAP: https://www.eecs.qmul.ac.uk/mmv/datasets/deap/download.html (accessed on 9 December 2017), DREAMER: https://zenodo.org/record/546113 (accessed on 11 August 2019), AMIGOS: http://www.eecs.qmul.ac.uk/mmv/datasets/amigos/download.html (accessed on 9 April 2020) and SEED: https://bcmi.sjtu.edu.cn/home/seed/seed.html (accessed on 12 August 2019).
